# Overexpression of *OsGF14f* Enhances Quantitative Leaf Blast and Bacterial Blight Resistance in Rice

**DOI:** 10.3390/ijms23137440

**Published:** 2022-07-04

**Authors:** Yamei Ma, Jianyuan Yang, Jingfang Dong, Shaohong Zhang, Wu Yang, Junliang Zhao, Tifeng Yang, Luo Chen, Lian Zhou, Jian Wang, Jiansong Chen, Wenhui Li, Wei Wu, Qing Liu, Bin Liu

**Affiliations:** 1 Rice Research Institute, Guangdong Academy of Agricultural Sciences, Guangzhou 510640, China; mayamei@gdaas.cn (Y.M.); dongjingfang@gdaas.cn (J.D.); szhanggz@tom.com (S.Z.); yangwu@gdaas.cn (W.Y.); zhao_junliang@gdaas.cn (J.Z.); yangtifeng@gdaas.cn (T.Y.); chenluo@gdaas.cn (L.C.); zhoulian@gdaas.cn (L.Z.); wjian@gdaas.cn (J.W.); chenjiansong@gdaas.cn (J.C.); liwenhui@gdaas.cn (W.L.); wuwei@gdaas.cn (W.W.); 2 Guangdong Key Laboratory of New Technology in Rice Breeding, Guangzhou 510640, China; 3 Guangdong Rice Engineering Laboratory, Guangzhou 510640, China; 4 Guangdong Key Laboratory of New Technology in Plant Protection, Plant Protection Research Institute, Guangdong Academy of Agricultural Sciences, Guangzhou 510640, China; yangjy@gdppri.com

**Keywords:** GF14f, leaf blast resistance, bacterial blight resistance, SA pathway

## Abstract

Although it is known that rice 14-3-3 family genes are involved in various defense responses, the functions of *OsGF14f* in response to diseases have not been reported. Here, we showed that the transcription of *OsGF14f* was significantly induced by leaf blast infection, and the overexpression of *OsGF14f* quantitatively enhanced resistance to leaf blast and bacterial blight in rice. Further analysis showed that the expression levels of salicylic acid (SA) pathway-associated genes (*PAL1*, *NH1*, *PR1a* and *PR10*) in the *OsGF14f*-overexpressing plants, were higher than those in wild-type plants after inoculation with the blast isolate (*Magnaporthe oryzae* Barr). In addition, the expression level of *OsGF14f* was significantly induced after SA treatment, and higher endogenous SA levels were observed in the *OsGF14f*-overexpressing plants compared with that in wild-type plants, especially after blast challenge. Taken together, these results suggest that *OsGF14f* positively regulates leaf blast and bacterial blight resistance in rice via the SA-dependent signaling pathway.

## 1. Introduction

As sessile organisms, plants are continuously subjected to various types of stresses such as pathogen attacks, insect herbivory, and environmental stresses [1]. These stresses have adverse effects on plant growth and seed production. To survive, plants have developed intricate mechanisms to efficiently perceive external signals and to tailor their responses to the precise environmental conditions encountered [1,2]. Many genes were reported as important regulators in various responses to stresses [2]. Among these genes, the genes encoding 14-3-3 proteins are modulated by several biotic and abiotic stresses [3,4].

14-3-3 proteins belong to a family of regulatory proteins that are uniquely eukaryotic, and evolutionarily conserved across all eukaryotes [5]. In plants, 14-3-3s have long been thought to play important roles in defense responses [6,7,8,9,10,11]. For example, early research indicated that *14-3-3* genes are differentially modulated during various disease defense responses [6,7,12]. Additionally, 14-3-3s are also involved in R-gene mediated plant disease resistance, as well as in reactive oxygen species (ROS)-mediated plant defense responses [9,13,14,15]. In addition, 14-3-3s have been implicated in programmed cell death (PCD) in tomatoes through directly interacting with mitogen-activated protein (MAP) kinase kinase kinase (MAPKKKa) and SIMKK2 [8,16]. Recently, 14-3-3 proteins have been reported to be the targets of bacterial effectors, and the interactions between 14-3-3 proteins and effectors affect bacterial virulence [10,17,18].

Rice (*Oryza sativa* L.) is the staple food for over half of the world population, but its production and quality are severely threatened by various diseases. Bacterial blight and blast, caused by the bacteria *Xathomonas oryzae* pv. *oryzae* (*Xoo*) and the fungus *Magnaporthe oryzae* Barr (*M. oryzae*), respectively, are the most destructive rice diseases, and can lead to a 30%-50% yield loss when these diseases become epidemic [19]. In our previous studies, we found that the overexpression of *OsGF14e* enhances resistance to panicle blast, whereas silencing *OsGF14e* results in increased susceptibility to panicle blast in rice [20]. *OsGF14b* positively regulates panicle blast resistance but negatively regulates leaf blast. Further results showed that *OsGF14b* is a target of WRKY71, which positively regulates rice resistance to panicle blast [21]. Moreover, a multi-omics study revealed that *OsGF14b* strongly activated the gibberellin, auxin and jasmonic acid signaling pathways during pathogen infection [22]. An earlier study demonstrated that *OsGF14f* was significantly up-regulated during the rice–*M. oryzae* and rice–*Xoo* interactions [4], suggesting *OsGF14f* may play important roles in biotic stresses in rice. However, all these suggested roles are based on its changes in expression level, and the actual functions of *OsGF14f* in biotic stresses in rice remain to be confirmed.

In the present study, the biological functions of *OsGF14f* in leaf blast and bacterial blight resistance and the underlying molecular mechanisms were investigated using *OsGF14f*-overexpressing rice plants and their wild-type plants. Our results suggest that *OsGF14f* functions as a positive regulator to modulate disease resistance in a quantitative manner, and *OsGF14f*-mediated disease resistance is associated with the activation of the SA signaling pathway.

## 2. Results

### 2.1. Expression Patterns of OsGF14f and Subcellular Localization of OsGF14f

**Figure 1:**
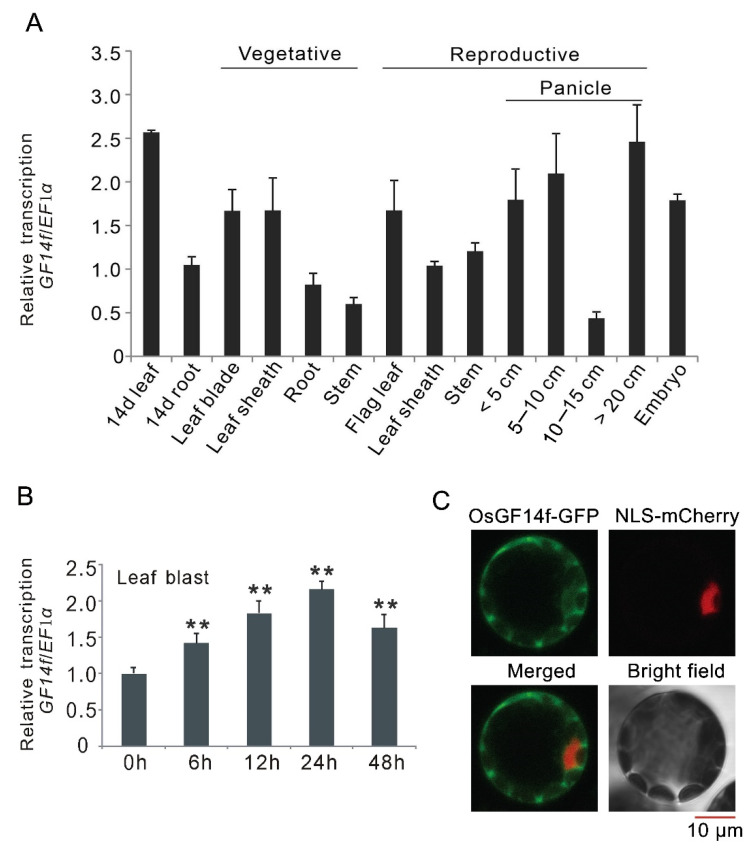
Expression patterns of *OsGF14f* and subcellular localization of OsGF14f. (**A**) Expression patterns of *OsGF14f* in different tissues of *Nipponbare* plants. (**B**) Quantitative RT-PCR analysis of the response of *OsGF14f* to leaf blast infection. The values 3 h, 6 h, 9 h, 12 h, 24 h and 48 h indicate the time after blast inoculation. ** *p* < 0.01. (**C**) Subcellular localization of OsGF14f. GF14f-GFP and the nuclear marker (NLS-mCherry) were co-transformed into *Arabidopsis* protoplasts. Fluorescence signal was detected by laser confocal microscopy after 16 h of transfection.

To provide insight into the function of *OsGF14f* during different physiological processes, tissue-specific expression pattern was examined in various tissues. As shown in Figure 1A, *OsGF14f* was ubiquitously expressed in all tested tissues, with the highest expression in 14-day-old leaf, and the lowest expression in 10-15 cm panicle. To understand the functions of *OsGF14f* in blast resistance, quantitative RT–PCR analysis was performed to monitor *OsGF14f* gene expression at 6, 12, 24 and 48 h after leaf blast inoculation. The qRT–PCR data showed that the transcription of *OsGF14f* was rapidly and significantly induced by the infection of the blast pathogen (Figure 1B). To examine the subcellular localization of OsGF14f, we transiently co-expressed a GF14f-GFP fusion construct with a nuclear marker (NLS-mCherry) in *Arabidopsis* protoplasts. As shown in Figure 1C, GF14f-GFP was mainly localized in the cytoplasm, with some occurrence in the nucleus (Figure 1C).

### 2.2. Overexpression of OsGF14f Enhances Resistance to Leaf Blast in Rice

**Figure 2:**
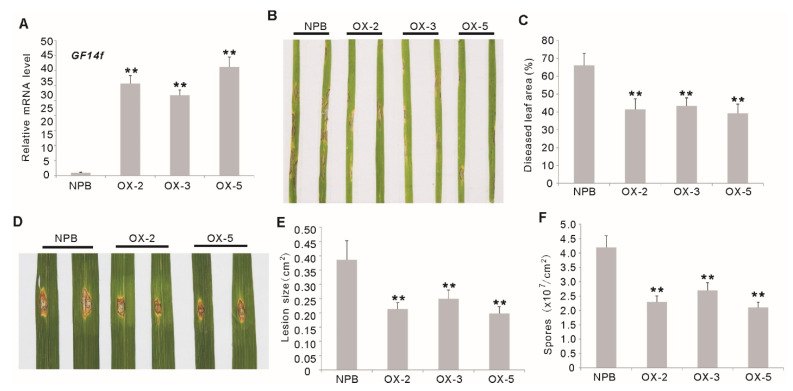
Disease phenotypes of the *OsGF14f*-overexpressing (*OXGF14f*) plants (OX-2, OX-3, OX-5) after leaf blast infection. (**A**) Transcription analysis of *OsGF14f* in the *OXGF14f* plants by quantitative RT-PCR. ** *p* < 0.01. (**B**) Disease phenotypes of the *OXGF14f* plants and *Nipponbare* plants after spraying inoculation with *M. oryzae* isolate GD08-T13 for six days. (**C**) Diseased leaf area (DLA) in the *OXGF14f* plants and *Nipponbare* plants after leaf blast inoculation for 6 days. Error bars indicate the SD from at least ten biological replicates. ** *p* < 0.01. (**D**) Disease phenotypes of the *OXGF14f* plants and *Nipponbare* plants after inoculation with *M. oryzae* isolate GD08-T13 using punch method at 6 weeks after sowing. (**E**) Relative lesion size in the *OXGF14f* plants and Nipponbare plants after leaf blast inoculation. Error bars indicate the SD from at least ten biological replicates. ** *p* < 0.01. (**F**) Numbers of spores produced on the *OXGF14f* plants and *Nipponbare* plants after punch inoculation. Error bars indicate the SD from three biological replicates. ** *p* < 0.01.

To confirm the function of *OsGF14f* in blast resistance in rice, transgenic rice plants constitutively overexpressing *OsGF14f* (*OXGF14f*), under the control of the CaMV 35S promoter, were produced in *Nipponbare*. Three independent homozygous lines (OX-2, OX-3 and OX-5), with increased transcription of *GF14f* were selected for disease evaluation (Figure 2A). We inoculated 2-week-old *OXGF14f* plants with the blast isolate GD08-T13, which showed strong virulence to *Nipponbare* plants, by the spray-inoculation method. The diseased leaf area ranged from 29.61% to 56.02% (average, 39.23%) in transgenic plants, significantly lower than that in wild-type plants (77.35%) (*p* < 0.01) (Figure 2B,C). To precisely quantify the *OsGF14f*-mediated quantitative blast resistance, we inoculated the *OXGF14f* plants with the same isolate by the punch method. Our results showed that the lesions on the leaves of *OXGF14f* plants were significantly smaller than that on the leaves of *Nipponbare* plants (*p* < 0.01) (Figure 2D,E). Moreover, the spore number on the leaves of *OXGF14f* plants was also significantly less than that on *Nipponbare* leaves (*p* < 0.01) (Figure 2F). These results suggest that the overexpression of *OsGF14f* can improve quantitative resistance against leaf blast in rice.

### 2.3. Overexpression of OsGF14f Enhances Resistance to Bacterial Blight in Rice

**Figure 3:**
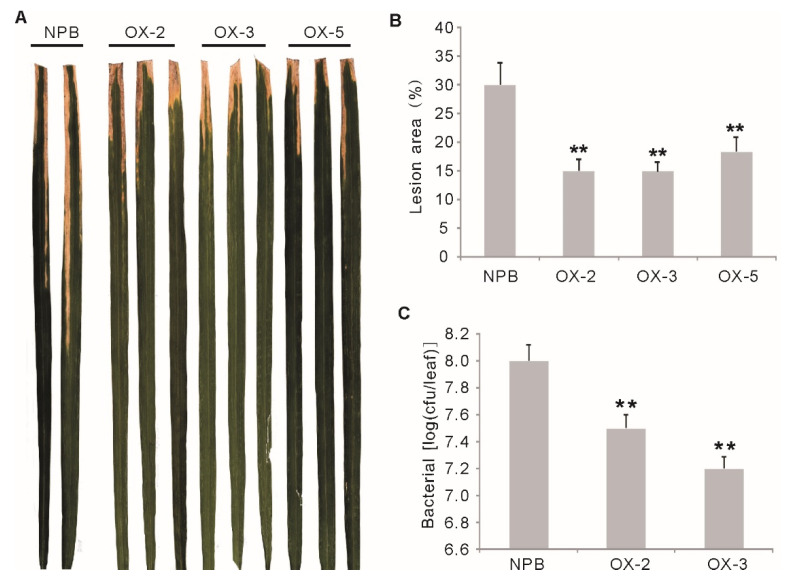
Disease phenotypes of the *OXGF14f* plants after bacterial blight infection. (**A**) Disease phenotypes of the *OXGF14f* plants and *Nipponbare* plants after Xoo inoculation. (**B**) Relative lesion area in the *OXGF14f* plants and control plants after *Xoo* inoculation. Error bars indicate the SD from at least ten biological replicates. ** *p* < 0.01. (**C**) The growth of the bacterial from *Xoo* race 4 in leaves of the *OXGF14f* plants and *Nipponbare* plants. Bacterial populations were determined from three leaves at 14 days after inoculation by counting colony-forming units (cfu). Similar results were obtained in two independent biological experiments. Error bars indicate the SD from six biological replicates. ** *p* < 0.01.

Bacterial blight caused by *Xanthomonas oryzae pv. oryzae (Xoo)*, is another devastating rice disease worldwide. Previous research has suggested that blast resistance and bacterial blight resistance might share common pathways to some extent [23]. To see if the *OsGF14f* gene also functions in bacterial blight resistance, we evaluated the bacterial blight resistance of the *OXGF14f* plants by inoculation with an isolate from Chinese *Xoo* race 4. The results showed that the *OXGF14f* plants showed significantly enhanced resistance to Chinese *Xoo* race 4 (*p* < 0.01) (Figure 3A), with the lesion area percentage ranging from 14.9 to 18.3% versus 30% for *Nipponbare* (Figure 3B). The growth rate of bacteria on the leaves of *OXGF14f* plants was much slower than that of the wild-type plants at 16 days after inoculation (Figure 3C). These results suggest that the overexpression of *OsGF14f* enhances quantitative resistance to bacterial blight in rice.

### 2.4. OsGF14f Positively Regulates the SA-Dependent Pathway Instead of JA Pathway

**Figure 4:**
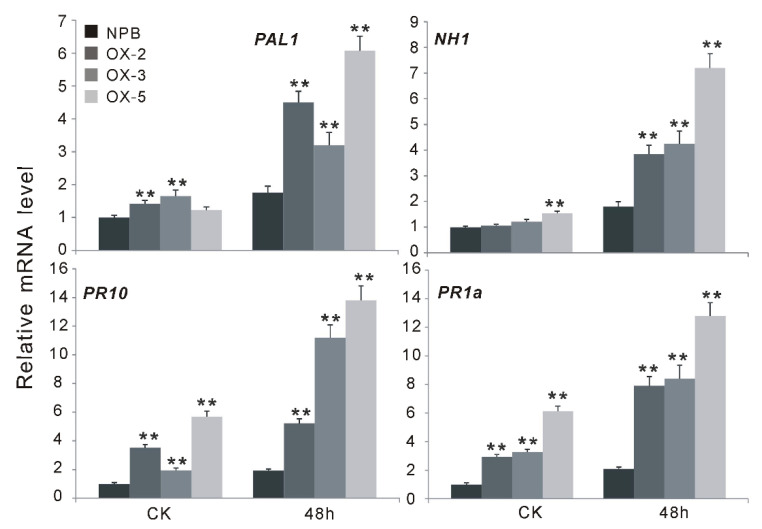
The expression changes of the genes associated with the salicylic acid (SA) signaling pathway before and after blast infection. Overexpression of *OsGF14f* significantly induced the expression of SA responsive genes and the SA synthesis-related genes. ** *p* < 0.01.

Jasmonic acid (JA) and salicylic acid (SA) are the major defense signaling compounds mediating disease resistance in plants [24]. To determine whether these signaling compounds are involved in *OsGF14f*-mediated defense responses, we analyzed the expression patterns of some well-characterized defense-related genes, which are associated with JA- or SA-dependent pathways, in both the *Nipponbare* and the *OsGF14f* transgenic plants before and after blast infection. Two *LOX* (lipoxygenase, *LOX1* and *LOX11*) genes and *AOS2* (allene oxide synthase 2), which are involved in the JA-dependent pathway, and two pathogenesis-related *PR* (*PR1a* and *PR10*) genes, *NHI* (Arabidopsis NPR1 homolog 1) and *PAL1* (phenylalanine ammonia-lyase), which are associated with the SA-dependent pathway [25] were selected for the study. The blast pathogen infection strongly induced the expression of *PAL1*, *NH1*, *PR1a* and *PR10*, both in *Nipponbare* and in *OsGF14f*-overexpressing plants (Figure 4). The expression levels of *PAL1*, *PR10* and *PR1a* were significantly higher in *OXGF14f* plants than in *Nipponbare* plants, either before or after infection (*p* < 0.01) (Figure 4). Although the expression level of *NH1* was not significantly changed in the transgenic plants compared to that in control plants before inoculation (Figure 4), its transcript was strongly induced in *OXGF14f* plants compared to that in control plants after inoculation (Figure 4). There was no obvious difference in the expression levels of *LOX1, LOX11* and *AOS2* between transgenic plants and wild-type plants, either before or after pathogen inoculation (Supplemental Figure S1). These results suggest that *OsGF14f*-mediated blast resistance may be involved in SA-dependent instead of JA-dependent signaling pathways.

### 2.5. OsGF14f Is Induced by SA Treatment and Overexpression of OsGF14f Can Increase Endogenous SA Levels

**Figure 5:**
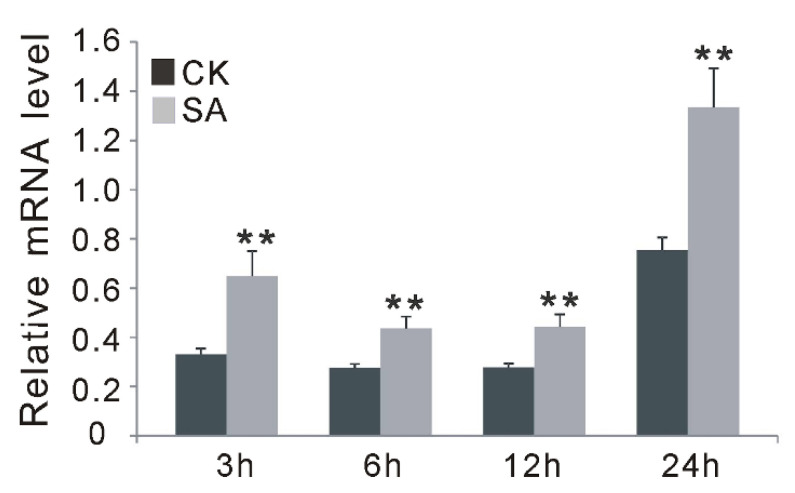
Time-course expression analysis of *OsGF14f* after SA and water (CK) treatments by quantitative RT-PCR analysis in *Nipponbare* plants. ** *p* < 0.01.

**Figure 6:**
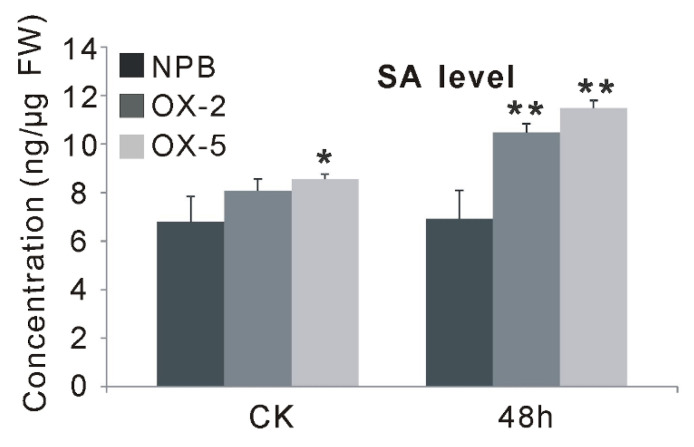
The SA levels of the *OXGF14f* and wild-type plants before and after blast infection. * *p* < 0.05, ** *p* < 0.01.

To further support the involvement of *OsGF14f* in SA-dependent signaling pathways, exogenous SA treatment was applied, and the expression changes of *OsGF14f* in wild-type plants at different time points (3, 6, 12 and 24 h) were monitored using qRT-PCR. Our results showed that the transcription levels of *OsGF14f* increased at all four time points after SA treatment in *Nipponbare*, with the peak at 24 h (Figure 5). In addition, we also observed that the endogenous SA level in *OXGF14f* plants was significantly increased after blast infection, while it remained at the same level in wild-type plants; and the SA level was significantly higher (*p* < 0.05) in *OXGF14f* plants than that in wild-type plants, both before and after blast infection (Figure 6). Together, these results suggest that *OsGF14f* positively regulates the SA-dependent pathway.

## 3. Discussion

### 3.1. OsGF14f Enhances Blast and Bacterial Blight Resistance in Rice in a Quantitative Manner

It is well-known that plants fight against blast invasion via two different resistance strategies: qualitative (complete) resistance, mediated by major disease resistance (*R*) genes, and quantitative (partial) resistance, contributed by multiple genes or quantitative trait loci (QTL) [26,27]. In most cases, qualitative resistance mediated by *R* genes is highly efficient, but it is race-specific and easily overcome, owing to the rapid evolution of pathogens [27,28]. In contrast, quantitative resistance conferred by quantitative trait loci (QTL) is presumably non-race-specific and is generally considered to be more broad-spectrum and durable under natural conditions [26]. Thus, quantitative resistance has been considered as a preferred strategy in disease control in rice [29]. Over the past two decades, numerous QTLs for quantitative blast and bacterial blight resistance have been identified. However, these sources have not been effectively used for the improvement of blast and bacterial blight resistance in rice because the genes of underlying resistance QTLs are unknown. Therefore, the identification and isolation of the genes of underlying resistance QTLs is the key for effective molecular breeding for quantitative disease resistance. In the present study, our results showed that enhanced disease resistance correlated with increasing transcription levels of *OsGF14f* (Figure 1, Figure 2 and Figure 3). Moreover, the *OsGF14f*-overexpressing rice plants displayed less diseased leaf area, smaller lesion size, fewer spores during blast infection, lower percent lesion length and fewer spores during bacterial blight infection compared with the wild-type plants (Figure 2 and Figure 3), exhibiting the nature of quantitative disease resistance. These results suggest that *OsGF14f* confers blast and bacterial blight resistance in a quantitative manner, and is a good target in molecular breeding for durable disease resistance in rice.

### 3.2. OsGF14f-Mediated Disease Resistance Is Involved in the SA-Dependent Pathway

SA is an archetypal defense hormone and its importance in the hard wiring of the plant innate immune system is well documented, particularly in the model plant *Arabidopsis* [30]. In this study, we showed that the expression levels of the defense-related genes, including *PAL1*, *NH1*, *PR1a* and *PR10* which are involved in the SA-dependent pathway, were significantly higher in *OsGF14f* transgenic plants than in *Nipponbare* plants, both before and after pathogen infection (Figure 4). Moreover, the transcript of *OsGF14f* was significantly induced by exogenous SA, and the endogenous SA levels were significantly higher (*p* < 0.05) in *OXGF14f* plants than those in wild-type *Nipponbare* plants, both before and after fungal infection (Figure 5 and Figure 6). These results suggest that *OsGF14f* mediated disease resistance is involved in the SA-dependent pathway. This is consistent with the previous findings, that the resistance to biotrophic and hemi-biotrophic pathogens is frequently controlled by the SA-dependent pathway [31]. We also found that the expression of *LOX1*, *LOX11* and *AOS2* is not changed between transgenic plants and wild-type plants, either before or after pathogen inoculation (Supplemental Figure S1), indicating that the JA-dependent pathway might function scarcely in *OsGF14f*-mediated disease resistance. Interestingly, in our previous study, we identified that *OsGF14b*, another member of the 14-3-3 family, was induced by blast infection and positively regulates panicle blast resistance in rice [20]. However, *OsGF14b*-mediated disease resistance is associated with the activation of the JA-dependent pathway and suppression of the SA-dependent pathway [21], different to the *OsGF14f*-mediated disease resistance pathway. These results suggest that there might be divergent functions and regulatory mechanisms in disease resistance among the members of the 14-3-3 family.

In conclusion, we have confirmed the functions of *OsGF14f* in response to blast and bacterial blight infection through gene expression analysis, a transgenic method and physiology analysis, and its possible regulatory mechanisms underlying these processes were also investigated. Our results indicate that *OsGF14f* positively regulates leaf blast and bacterial blight resistance in rice. *OsGF14f*-mediated disease resistance is dependent on the SA signaling pathway. Since blast and bacterial blight are two of the most devastating diseases in rice, and quantitative disease resistance is more durable, *OsGF14f* is a good target in rice improvement for disease resistance.

## 4. Materials and Methods

### 4.1. Plant Materials and Pathogens

The *OsGF14f*-overexpressing rice plants and their wild-type plants, *Nipponbare*, were used in this study. Rice seeds were surface-sterilized and transferred to Murashige and Skoog medium [32] and incubated in a growth chamber under light of 200 μmol/m^2^/s with a 12 h photoperiod at 26 °C. After germination, rice seedlings were transplanted into soil and kept in a greenhouse. Blast (*M. oryzae*) isolate GD08-T13 collected from Guangdong, China, was used for rice blast inoculation, and Chinese *Xoo* race 4 isolate collected from Guangdong, China, was used for bacterial blight inoculation.

### 4.2. Pathogen Inoculation and Evaluation of Disease Resistance

The inoculum of *M. oryzae* isolate GD08-T13 was prepared as described by Liu et al. (2018) [33]. The revived isolate GD08-T13 was cultured on prune agar plates (three pieces prune, 5 g lactose, 1 g yeast extract, and 20 g agar bar in 1 L, pH of 6–6.5, autoclaved) for 7 d at room temperature, and mycelial growth was scraped with a sterilized spatula and exposed to fluorescent light for 4–5 days to induce sporulation. Spores were collected and suspended in water to reach a concentration of 1 × 10^6^ mL^−1^. To examine the effect of rice blast inoculation on the expression of *OsGF14f*, conidia of GD08-T13 were washed and suspended at 1 × 10^6^ cells mL^−1^ in sterile water, and sprayed on the 14-day-old *Nipponbare* plants, which were then incubated at 26 °C in a growth chamber (16,000 Lux and 100% relative humidity) in the dark for 24 h, followed by 12 h light and 12 h dark cycles.

Two inoculation methods were used in evaluation of blast resistance in this study. For spray inoculation, two-week-old rice seedlings were sprayed with a spore suspension of GD08-T13 (1 × 10^6^ spores/mL) containing 0.05% Tween-20. Inoculated plants were maintained in the same growth chamber used for transcription analysis at 26 °C, 16,000 Lux and 100% relative humidity in the dark for 24 h. Subsequently, the growth chamber was set to a photoperiod of 2 h light and 12 h dark at 26 °C and 100% relative humidity. Disease was assessed 6 d after inoculation by measuring the diseased leaf area. For punch inoculation, six-week-old plants were punched as described previously [34,35]. Briefly, 5 μL of spore suspension of GD08-T13 (5 × 10^5^ spores/mL) containing 0.05% Tween-20 was added to the press-injured spots on fully expanded rice leaves; the inoculated spots were wrapped with transparent Scotch tape, and inoculated leaves were photographed at 14 days after inoculation, and lesion size was measured using ImageJ (http://rsbweb.nih.gov/ij/, accessed on 8 July 2021). For determination of in planta sporulation after punch inoculation, leaf strips containing a lesion spot were excised and submerged in 100 μL of distilled water, in a 1.5 mL microcentrifuge tube. After the suspension was vigorously mixed, spores were counted with a microscope. To evaluate bacterial blight disease, plants were inoculated with *Chinese Xoo race 4* (OD_600_ = 0.3) isolate at the booting stage by the leaf-clipping method [36]. Disease was scored by measuring the percent lesion length (lesion length/leaf length) at 12 d after inoculation.

### 4.3. Plasmid Construction and Rice Transformation

To generate the overexpression vector of *OsGF14f*, we obtained the full-length coding region of the gene from *Nipponbare* leaf cDNA by PCR using primers *GF14f*-OE-F/R (Supplemental Table S1), and cloned the fragment into the PHQSN (modified from pCAMBIA1390), harboring a CaMV 35S promoter. The plasmid was transferred into the calli induced from the immature seeds of *Nipponbare*, using the *Agrobacterium tumefaciens*-mediated rice transformation method as previously described [37].

### 4.4. Subcellular Localization

The full-length cDNA of *OsGF14f* was constructed into the pCAMBIA1300-GFP to generate *pUBQ10: OsGF14f-GFP* construct. The OsGF14f-GFP and NLS-mCherry plasmid were co-transformed into Arabidopsis protoplasts for transient expression following previously described method [38]. After incubation in darkness for 16 h, images were captured using a confocal fluorescence microscope (LSM 710).

### 4.5. Gene Expression Analysis

Total RNA was extracted from rice tissues using TRIzol reagent (Invitrogen, Carlsbad, CA, USA), and was treated with DNaseI (Takara, Dalian, China) to remove genomic DNA contamination. The first strand of cDNA was synthesized from 1 μg of total RNA using the primescript^TM^ RT reagent kit (Takara, Dalian, China) according to the manufacturer’s instructions. Quantitative PCR was performed using SYBR Premix ExTaq^TM^ (Takara, Dalian, China) on a CFX Connect real-time PCR detection system (Bio-Rad, Hercules, CA, USA), and data were analyzed using Bio-Rad CFX Manager 3.1 (Bio-Rad, Hercules, CA, USA). *EF1a* was used as the internal control. Gene-specific primers that were used are listed in Supplemental Table S1.

### 4.6. SA Treatment and Measurement

To examine the effect of rice blast inoculation on the expression of *OsGF14f*, two-week-old rice seedlings were sprinkled with 100 μM hormone solution, and sampled at 3 h, 6 h, 12 h and 24 h after treatment.

The hormones SA were extracted from the rice leaf [39], and then 10 μL of SA sample solution was injected onto a C18 column (AQUITY UPLC BEH 130, 1.7 μm, 2.1 by 100 mm, Waters) at a flow rate of 0.1 mL/min, and the column was maintained at 30 °C. The sample solution was separated by reversed-phase ultra-fast LC (Shimadzu, Kyoto, Japan) with a multi-step linear gradient elution over 30 min. The eluate was then introduced into the electrospray ion source of a tandem triple quadrupole MS analyzer (API4000, AB SCIEX, Foster City, CA, USA), and the SA compound was quantified in the multiple reaction monitoring (MRM) mode using optimized MS/MS conditions, which are listed in Supplemental Table S2. The Analyst 1.5.2 software (AB SCIEX, Foster City, CA, USA) was used to control the instrument, and to acquire and process all of the MS data.

### 4.7. Statistical Analysis

All the graphic data are presented as mean  ±  SD. Student’s *t*-test was used to test the significance of difference: * and ** indicate a significant difference at *p*  <  0.05 and *p*  <  0.01, respectively.

## Data Availability

Not applicable.

## Supplementary Materials

The following supporting information can be downloaded at: https://www.mdpi.com/article/10.3390/ijms23137440/s1.
