# Supplementary material for: Overexpression of OsGF14f Enhances Quantitative Leaf Blast and Bacterial Blight Resistance in Rice
Source: Int J Mol Sci. 2022 Jul 4;23(13):7440. doi: 10.3390/ijms23137440 (PMC9266906; doi:10.3390/ijms23137440)
Supplement: Supplementary file 1 [file ijms-23-07440-s001.zip › ijms-1794296-supplementary.pdf]

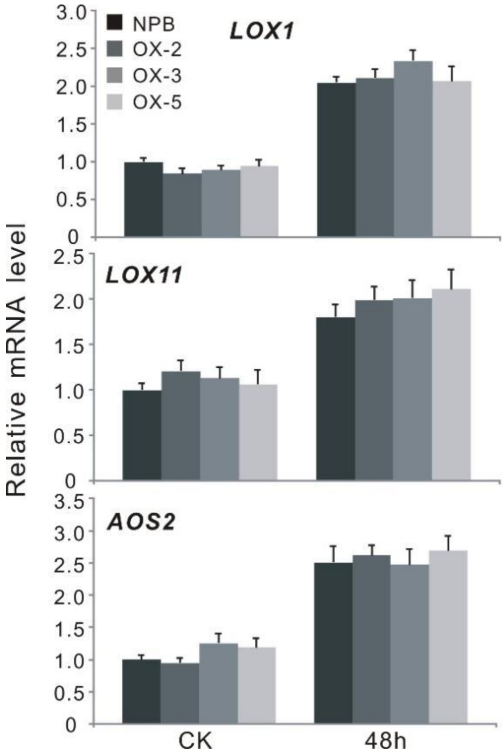

Figure S1. The expression patterns of three defense-related genes involved in jasmonic acid (JA)-dependent pathway before blast infection (CK) and at 48 h after infection with *M. oryzae*.

Table S1. Primers used for vector construction and Quantitative RT-PCR.

Gene	Forward primer (5'-3')	Reverse primer (5'-3')
GF14f-OE	ACAAGGTCAGACTAAGTTTGCG	TGACATACGCAGCCCTAATCTT
LOX1	GGTGCAGCACCATCGAGCAG	TACGTGTCCTGCTTCATCGTCC
LOX11	CGACCAAGAACAGACGACCGA	TGCCATCCATGTGCGAGTACCT
AOS2	CAATACGTGTACTGGTCGAATGG	AAGGTGTCGTACCGGAGGAA
PR10	CCCTGCCGAATACGCCTAA	CTCAAACGCCACGAGAATTTG
NH1	CACGCCTAAGCCTCGGATTA	TCAGTGAGCAGCATCCTGACTAG
PAL1	AGCACATCTTGGAGGGAAGCT	GCGCGGATAACCTCAATTTG
PR1a	CGTCTTCATCACCTGCAACTACTC	CATGCATAAACACGTAGCATAGCA
GF14f	ACAAGGTCAGACTAAGTTTGCGTT	GAATTCGACCATTTCCTCGTAAC
EF1α	TTTCACTCTTGGTGTGAAGCAGAT	GACTTCCTTCACGATTTCATCGTAA

Table S2. Characteristic fragment ions of the SA standard and its optimized MS/MS conditions.

		**Transition1**			**Transition2**		
**Analyte**	**ESI mode**	Quantitative ion	Collision energy/ev	Declustering potential/ev	Quantitative ion	Collision energy/ev	Declustering potential/ev
**SA**	ESI-	136.9>65.0	-40	-40	136.9>92.8	-32	-40
